# Maternal Hydroxytyrosol Supplementation Enhances Antioxidant Capacity and Immunometabolic Adaptations in Nutrient-Restricted Beef Cows and Their Offspring

**DOI:** 10.3390/antiox14091097

**Published:** 2025-09-08

**Authors:** Nieves Escalera-Moreno, Javier Álvarez-Rodríguez, Leire López de Armentia, Alba Macià, Maria José Martín-Alonso, Ester Molina, Daniel Villalba, Albina Sanz, Beatriz Serrano-Pérez

**Affiliations:** 1 Animal Science Department, University of Lleida, Av. Rovira Roure 191, 25198 Lleida, Spain; mariajose.martin@udl.cat (M.J.M.-A.); ester.molina@udl.cat (E.M.); daniel.villalba@udl.cat (D.V.); 2 Departamento de Producción Animal y Ciencia de los Alimentos, Escuela Politécnica Superior de Huesca, Universidad de Zaragoza-CITA-IA2, Carretera de Cuarte s/n, 22071 Huesca, Spain; javier.alvarezr@unizar.es; 3 Centro de Investigación y Tecnología Agroalimentaria de Aragón (CITA) (IA2-UNIZAR), Avda. Montañana 930, 50059 Zaragoza, Spain; llopezdearmentia@cita-aragon.es (L.L.d.A.); asanz@aragon.es (A.S.); 4 Food Technology, Engineering and Science Department, XaRTA-TPV, University of Lleida, Av. Rovira Roure 191, 25198 Lleida, Spain; alba.macia@udl.cat; 5 AGROTECNIO-CERCA Center, University of Lleida, 25198 Lleida, Spain

**Keywords:** energy metabolism, fetal programming, immune response, late gestation, maternal nutrition, neonatal calves, oxidative stress

## Abstract

The impact of maternal dietary restriction and hydroxytyrosol (HT) supplementation during the last third of gestation on plasma malondialdehyde (MDA) concentration, total antioxidant capacity (ABTS assay), and peripheral blood gene expression related to antioxidant defence, immune response, and energy metabolism was evaluated in beef cows and calves. Two feeding treatments in late gestation (T100% vs. T60% of nutrient requirements) and two HT levels (Control vs. HT at 180 mg/kg of diet) were evaluated during gestation (*n* = 46 cows) and lactation (*n* = 37 cows and calves). In pregnant cows, undernutrition led to inhibition of glucose oxidation (*PDK4*), decreased lipid synthesis (*HMGCS1* and *SCD*) and TLR signalling; T60% cows showed higher plasma MDA (*p* < 0.05) with no positive effect of HT on antioxidant capacity. Contrarily, during lactation, earlier HT supplementation upregulated antioxidant capacity and modulated antioxidant gene expression (*p* < 0.05). In calves, there was an increase in *SOD1*, *CAT*, and *GPX1*, especially in the T60%-HT group (*p* < 0.05). Interestingly, HT supplementation increased glucose transport (*SLC2A1*/*GLUT1*) during pregnancy and lactation (*p* < 0.05). However, it caused different effects on immunometabolic regulation in both dams and calves, depending on maternal diet. Overall, maternal HT supplementation under restricted nutritional conditions promoted postpartum antioxidant capacity and modulated immune and metabolic gene expression in cows and calves.

## 1. Introduction

The main challenges in beef cattle production systems are improving productivity and maximizing nutrient efficiency to minimize feed costs and reduce environmental impact. However, minimizing feed costs through dietary restrictions or the use of low-quality pasture resources can negatively impact reproduction [1]. In cattle, a high metabolic challenge during gestation affects cows’ ability to physiologically adapt to the homeorhetic demands associated with fetal growth, parturition, and lactogenesis. Consequently, challenged cows may develop metabolic stress, a condition characterized by excessive lipomobilization, immune dysfunction, inflammation, and oxidative stress, resulting from intense catabolic responses [2,3]. Metabolic adaptations necessary for lactation begin at the end of gestation, a critical period that not only influences maternal well-being but also the development and postnatal health of calves. The onset of lactation dramatically alters the metabolism of many maternal organs so that the mammary gland is supplied with nutrients necessary for the synthesis of milk.

Metabolic reprogramming reflects cell responses to critical changes in the environment that govern the nature of the immune response [4]. Immune cells possess signalling pathways that detect nutritional and energetic status, adjusting metabolism towards anabolic pathways, which are more antigenic or immunogenic, or catabolic pathways, which are more tolerogenic, respectively [5,6]. Enhanced glycolysis is a metabolic change in immune cells that have undergone rapid activation to carry out their particular effector functions in response to the stimulation of pattern recognition receptors, cytokine receptors, or antigen receptors (e.g., Toll-like receptors (TLRs)), which involves nuclear factor kappa B (NF-κB) activation with a more pro-inflammatory effector phenotype. Inflammatory stimuli in both innate and adaptive immune systems also trigger the fatty acid synthesis pathway that allows cells to generate lipids that are necessary for cellular growth, membrane biosynthesis and proliferation. On the contrary, cells involved in the resolution of immune responses and inflammation are adapted to function and slow growth in tissue environments where nutrients are more limiting and efficiency is crucial. Oxidative phosphorylation and a reliance on fatty acid oxidation have been observed in non-inflammatory and regulatory/tolerogenic cells with increased cellular lifespans [7]. However, recent studies have highlighted the heterogeneity in activation states [8,9]. Oxidative stress, proposed as a link between the metabolic and immune systems of cows, arises from an increase in catabolic pathways generating energy from lipids and amino acids, leading to lipid peroxidation [6,10].

Furthermore, maternal nutritional factors, particularly during late gestation, have been shown to significantly impact fetal development, endocrine regulation, and overall calf physiology [11,12,13], even when dietary restriction occurs early in pregnancy [14,15]. The first month of life in calves represents a critical period characterized by intense redox, immunological, and metabolic changes that impact their development and survival. As in cows, redox balance is crucial for the prevention of oxidative stress. Metabolic demands and the quality of the diet influence the animal antioxidant capacity [16]. Immunologically, calves initially rely on passive immunity acquired through maternal colostrum. However, during this period, the progressive activation of their own immune system begins [17]. They become vulnerable to various diseases due to immunosuppressive or inflammatory responses, as a result of the immaturity of their immune systems, when facing environmental challenges or pathogens [18]. Metabolically, calves undergo a significant transition as they adapt to a milk-based diet, which involves changes in carbohydrate and lipid metabolism. This phase is essential for establishing a solid metabolic foundation and healthy growth [19,20].

Among the strategies to mitigate the effects of undernutrition is the supplementation of antioxidants in the prepartum diet [21,22]. These compounds, present in many foods, may help neutralize free radicals and reduce oxidative stress in undernourished cows. Specifically, the use of phenolic compounds, such as hydroxytyrosol (HT), a natural compound that can be found in olive leaves [23], has demonstrated remarkable antioxidant activity in animal plasma, acting as an effective neutralizer of reactive oxygen species (ROS). Its catechol-like structure enables it to donate hydrogen atoms that stabilize free radicals present in the plasma milieu, thereby reducing lipid peroxidation and oxidative damage to plasma proteins. Furthermore, its ability to chelate metal ions, such as Fe^2+^ and Cu^2+^, limits metal-catalyzed prooxidant reactions [24]. Compared to other popular antioxidants, HT exhibits greater antioxidant activity [25] and demonstrates rapid absorption into the bloodstream, reaching detectable plasma concentrations within 5 to 10 min of ingestion [26]. Although current evidence on the use of HT in cattle is limited, its effects have been positively documented. In swine, maternal supplementation with HT during gestation improves fetal energy metabolism, as well as antioxidant and immune capacity, by modifying the lipid composition of tissues, protecting polyunsaturated fatty acids (PUFAs) from oxidation, and modulating the inflammatory balance during gestation [27,28].

This study evaluated the effects of undernutrition and supplementation with HT during the last third of gestation on malondialdehyde (MDA) concentration, total antioxidant capacity (ABTS assay) in blood plasma, and the expression of genes involved in antioxidant status, immune regulation, and metabolism pathways in cows during late pregnancy, as well as in cows and their respective calves after parturition.

## 2. Materials and Methods

### 2.1. Animal Ethics

The experiment was conducted in accordance with the requirements of the Spanish Policy for Animal Protection RD 53/2013 (BOE, 2013), which meets the European Union Directive 2010/63/EU on the protection of animals used for experimental and other scientific purposes. In addition, the in-house Ethical Committee of the ‘Centro de Investigación y Tecnología Agroalimentaria de Aragón (CITA)’ approved the animal procedure protocol (protocol number CEEA-04 2021-09).

### 2.2. Animals and Husbandry

The experiment was conducted at the experimental facilities of La Garcipollera Research Station in the mountain area of the central Pyrenees (northeastern Spain, 945 m a.s.l.), in Aragón (northeastern Spain), from November 2021 to March 2022. Cows were recruited from a large experimental suckler cattle herd composed exclusively of beef breeds, which were evenly represented. A full description of the animals included in this study can be found in [29]. The study animal sample comprised 46 prepartum cows (T100%-CTROL, *n* = 11; T100%-HT, *n* = 10; T60%-CTROL, *n* = 14; T60%-HT, *n* = 11) and 37 postpartum cows (aged 6.7 ± 1.5 years and initial body weight of 672 ± 48.5 kg; means ± standard deviations) with their nursing calves (T100%-CTROL, *n* = 10; T100%-HT, *n* = 9; T60%-CTROL, *n* = 9; T60%-HT, *n* = 9).

### 2.3. Dietary Treatments and Performance Recordings

Briefly, at 28 ± 0.7 weeks of gestation (twelve weeks before calving), healthy dams, free from any reproductive and clinical disease, were stratified according to expected calving date and age and then randomly allocated into 4 groups based on the starting date of gestation, within a 2 × 2 factorial design to evaluate the effects of feeding level. The groups included a diet that met 100% of nutrient requirements versus one that met only 60% (T100% vs. T60%) and HT supplementation as a total mixed ration (TMR), i.e., 0 mg HT/kg of TMR (CTROL) vs. 180 mg HT/kg of TMR (HT), added in the form of mash (100 g of HT with a 10% purity/per L of solution, and 18 L of solution per ton of diet) (Econatur, La Carlota, Córdoba, España). The restricted feeding level was based on commercial winter season practices [1] for pregnant cows, while the HT dose was selected based on health claims and no adverse effects in other animal species [30].

Feed was provided at 08:00 a.m., and the cows were tied up for a maximum of 3 h until they finished the assigned amount of feed (10.5 kg/day to T100% or 7.0 kg/day to T60%). The TMR was formulated to meet the nutritional requirements of late-gestation dairy cows and was offered as a mash. A detailed composition of the TMR ingredients, nutrient supply, and energy value is provided in the Supplementary Materials. The TMR composition was identical across the dietary treatments, except for the inclusion of HT. According to the INRA nutritive value calculations [31], the estimated energy value was 1240 kcal of Net Energy for lactation/kg of DM. At all times, the cows had additional access to mineral blocks, which were licked at an individual rate of approximately 75–80 g/day.

To ensure maximal uniformity, 37 pairs of cows and calves born consecutively from unassisted (eutocic) parturitions were selected. After calving, the cows were fed to cover 100% of their postpartum requirements and did not receive any type of polyphenol supplementation. The lactation diet had the same feed composition as the one used during gestation, but all dietary treatments were provided with 10.5 kg of TMR/day. All the cows and calves were loose-housed, with straw as indoor bedding and outdoor access. Each dietary treatment was replicated twice, with a total of eight group pens. The calves were nursed by the cows twice daily (two 30 min periods at 7:00 and 14:00 h). The dams were weighed at weeks −12, −9, and −3 before calving, just after calving, and at week 5 postpartum. The average daily gain (ADG) during the last stages of pregnancy and after parturition was calculated by linear regression.

### 2.4. Blood and Colostrum Sample Collection

**Figure 1:**
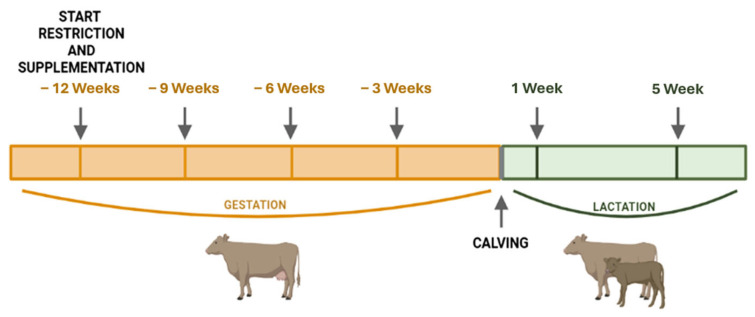
Schedule of blood sample collection dates before and after calving (created with [32]).

Samples were collected at weeks −9, −6, and −3 before calving from the pregnant cows, and at weeks 1 and 5 after calving from both the cows and their nursed calves (Figure 1). Two blood samples were individually collected from each cow through coccygeal puncture, and from each calf through jugular vein puncture, within a time frame between 8:00 a.m. and 10:00 a.m. The first blood sample was collected in heparin tubes for MDA and ABTS analysis. These samples were immediately centrifuged (2500 × *g* for 10 min) to obtain the plasma and then stored at −20 °C until analysis. The second blood sample was collected into Tempus^TM^ Blood RNA Tubes (Applied Biosystems, Foster City, CA, USA) for whole-blood gene expression analysis, according to the manufacturer’s instructions. These samples were shaken after sample collection and then stored at −20 °C until use. For the analysis of HT metabolites, serum and colostrum samples were collected in a selection of 24 dams (6 dams/group). Blood samples were collected in empty tubes at weeks −12 and −3, and colostrum samples were manually collected during the first 12 h after calving, frozen at −20 °C, and subsequently lyophilized.

### 2.5. Sample Analysis

#### 2.5.1. Determination of Malondialdehyde and Total Antioxidant Activity (ABTS Assay) in Plasma

MDA and ABTS (2,2′-azino-bis(3-ethylbenzothiazoline-6-sulfonic acid)) were analysed as pro- and anti-oxidative markers in plasma, respectively. The MDA levels were measured using a gas chromatography method that separately quantifies free MDA (FMDA) and protein-bound MDA (PBMDA) to ultimately determine total MDA. This method has been previously validated in bovine plasma [33]. The ABTS radical cation (μmol Trolox-eq./mL) was obtained by diluting the plasma to 1/60, mixing it with the ABTS solution, and subsequently calibrating it with Trolox as the reference standard, as a proven method to measure antioxidant capacity in the ruminant plasma [34]. Twelve replicates of each extract were prepared [35]. To minimize inter-operator variability, all assays were performed by the same trained technician under standardized laboratory conditions.

#### 2.5.2. HT Metabolite Analysis

The presence of specific phase II metabolites was assessed in both plasma and colostrum matrices. Among the potential metabolic products of HT, HT sulphate (HTS) and alcohol homovanillic sulphate (AHVS) were selected as compliance signature biomarkers, based on their specificity, detectability, and established role as metabolites of HT in mammalian systems. The tentative quantification of these phenolic metabolites was carried out by UPLC coupled to tandem mass spectrometry (MS/MS) [36], reaching a detection limit of 30 ng/mL and 15 ng/mL for HTS and AHVS, respectively.

#### 2.5.3. RNA Extraction, cDNA Synthesis, and qPCR Analysis

Total RNA was extracted from Tempus^TM^ Blood RNA Tubes (Applied Biosystems, Foster City, CA, USA) with a Tempus^TM^ Spin RNA Isolation Kit (Applied Biosystems, Foster City, CA, USA) according to the manufacturer’s recommendations. The concentration and purity of the total RNA were determined spectrophotometrically using a UV/Vis Implen NanoPhotometer N50 (Thermo Scientific, Waltham, MA, USA). The samples were treated with RNase-free DNase I (Thermo Scientific, Waltham, MA, USA) to eliminate contaminating genomic DNA. First-strand cDNA synthesis was performed with 0.3 µg of total RNA and random hexamer primers using the Maxima RevertAid H Minus First Strand cDNA Synthesis Kit (Thermo Scientific, Waltham, MA, USA) according to the manufacturer’s recommendations. The samples were stored at −80 °C for subsequent gene expression analysis.

To prevent genomic contamination, whenever possible, the primers were designed to span an intron. A standard curve was created for each gene by amplifying serial dilutions of a control cDNA to ensure linearity between the initial template concentration and the cycle threshold (Ct) values. Amplification was performed in a QuantStudio™ 7 Flex Real-Time PCR System sequence detector (Applied Biosystems, Foster City, CA, USA). The qPCR conditions included an initial activation and denaturation step of 10 min at 95 °C, followed by 40 cycles of 10 s at 95 °C and 1 min at 60 °C.

Messenger RNA expression was determined by qPCR. Genes selected for transcript analysis were those involved in antioxidant status (superoxide dismutase 1 (*SOD1*), superoxide dismutase 2 (*SOD2*), catalase (*CAT*), glutathione peroxidase 1 (*GPX1*), nuclear factor erythroid 2-related factor 2 (*NRF2*)), immune regulation (5-lipoxygenase (*ALOX5*), tumor necrosis factor alpha (*TNFA*), nuclear factor kappa B (*NFKB*), toll-like receptor 4 (*TLR4*), peroxisome proliferator-activated receptor delta (*PPARD*)) and energy metabolism (pyruvate dehydrogenase kinase isoenzyme 4 (*PDK4*), the gene encoding solute carrier family 2 member 1 (*SLC2A1*/*GLUT1*), insulin-like growth factor 1 receptor (*IGF1R*), acyl-CoA dehydrogenase (*ACADVL*), stearoyl-CoA desaturase (*SCD*), fatty acid synthase (*FASN*), sterol regulatory element-binding protein 1 (*SREBF1*), 3-hydroxy-3-methylglutaryl-CoA synthase 1 (*HMGCS1*)). The expression levels of the target gene were normalized to those of the combination of the two most stable reference genes (*ACTB* and *RPL19*), which were validated using NormFinder [37]. The sequences and sources of the primers are shown in Table 1. PCR reactions were run using 3 μL of 300-fold diluted cDNA as the template in a total volume of 8 μL. The reaction mixture contained 2.5 μL of PowerTrack SYBR Green Master Mix (Thermo Scientific, Waltham, MA, USA), 2 μL of H_2_O, and forward and reverse primer concentrations, as reported in Table 1 (125 nM–400 nM). Each measurement was carried out in triplicate, and the average was used to calculate the relative gene amount. Data were normalized and analysed with the 2^−ΔΔCt^ method using the mean Ct value obtained for the two reference genes and the Ct values for each sample.

### 2.6. Data Analysis

The data obtained were analysed using JMP Pro version 18 software (SAS Institute Inc., Cary, NC, USA). Blood MDA concentration, total antioxidant capacity (ABTS assay), and gene expression at weeks −9, −6 and −3 before calving in the pregnant cows, and at weeks 1 and 5 after calving in the cows and their nursed calves, were analysed in separate data sets by means of mixed models with repeated measures. The fixed effects included feeding level (T100% vs. T60%), HT supplementation (CTROL vs. HT), sampling week (weeks −9, −6, and −3 before calving, or weeks 1 and 5 after calving), as well as their interactions. Each cow was considered as the experimental unit and included as a random effect to account for individual variability among animals. Starting body weight at week −12 before calving was considered as a covariate in all models. The interactions between fixed effects are only reported in Section 3 if they are significant (*p* < 0.05). Blood gene expression data were log-transformed to meet the assumption of normality and homoscedasticity. Least square means and their standard errors are presented. Normalized gene expression values were statistically analysed in log form but expressed as relative quantification (RQ). The separation of means was carried out with Tukey’s test. Chi-square tests were used to assess associations between HT metabolite detection in serum (weeks −12 and −3) and colostrum (<12 h parturition) and factors of interest (feeding level and HT supplementation). The level of significance was set at *p*-value < 0.05.

## 3. Results

### 3.1. Cow Performance and Circulating HT Metabolites

During the last third of pregnancy, the feeding level affected (*p* < 0.001) the cow ADG, such that the T100% cows showed greater ADG during the prepartum period than the T60% cows (Table 2). Consequently, the BW after calving was greater (*p* < 0.001) in the T100% cows than in the T60% cows. The loss of ADG during the first month of lactation was greater (*p* < 0.05) in the T100% cows than in the T60% cows. However, the gestation dietary HT supplementation did not affect (*p* > 0.10) the cow ADG either during the last third of pregnancy or the first month of lactation (Table 2).

### 3.2. MDA Concentration and Total Antioxidant Capacity in Plasma

**Figure 2:**
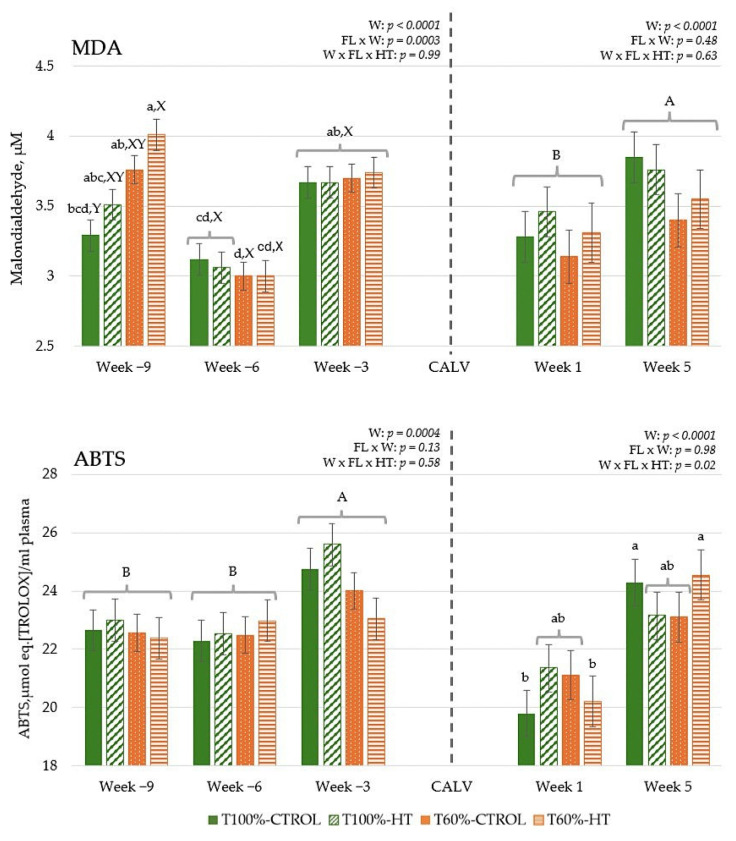
Interaction between feeding level (FL) and hydroxytyrosol (HT) supplementation on total plasma malondialdehyde (MDA) concentration and total antioxidant capacity (ABTS assay) at given weeks (W) relative to calving (CALV) in cows during gestation (T100%-CTROL, *n* = 11; T100%-HT, *n* = 10; T60%-CTROL, *n* = 14; T60%-HT, *n* = 11) and during lactation (T100%-CTROL, *n* = 10; T100%-HT, *n* = 9; T60%-CTROL, *n* = 9; T60%-HT, *n* = 9). Means with different letters differ at *p* < 0.05. If only the week had a significant effect, its letters (A, B) are shown. If there are any other significant effects, letters from the triple interaction (a, b, c, d) are used, and if any interactions between feeding level or supplementation with week occur, differences within the same week (X, Y) are also included. Groups represented with the same letters between gestation and lactation are not related.

During gestation, the total MDA concentration was higher (*p* < 0.001) at week −9 in T60%-HT than in T100%-CTROL, although no differences were observed between the treatments in the rest of the sampling weeks (Figure 2). MDA values decreased at week −6 and increased again at week −3 (*p* < 0.001). After calving, total MDA increased as lactation progressed and was significantly higher at week 5 compared to week 1 (*p* < 0.05) (Figure 2). During gestation, the levels of ABTS were significantly increased at week −3 compared to weeks −6 and −9 (*p* < 0.01). During lactation, total antioxidant capacity was significantly increased for T100%-CTROL and T60%-HT at week 5 compared to the other groups (*p* < 0.05). Total antioxidant capacity was also significantly increased at week 5 as compared to week 1 (*p* < 0.001) (Figure 2).

**Figure 3:**
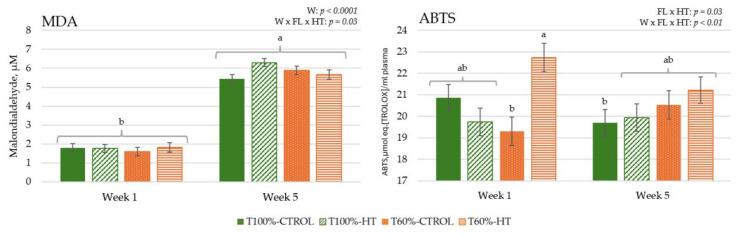
Interaction between maternal feeding level (FL) and hydroxytyrosol (HT) supplementation on total plasma malondialdehyde (MDA) concentration and total antioxidant capacity (ABTS assay) in calves at given weeks (W) relative to birth (T100%-CTROL, *n* = 10; T100%-HT, *n* = 9; T60%-CTROL, *n* = 9; T60%-HT, *n* = 9). Means with different letters differ at *p* < 0.05. If there are any other significant effects, letters from the triple interaction (a, b) are used.

In the calves, MDA levels were also significantly increased at week 5 as compared to week 1 (*p* < 0.001) (Figure 3). Higher total antioxidant capacity was observed in the calves from T60%-HT at week 1 dams compared to the T60%-CTROL group at the same week and the T100%-CTROL group at week 5 (*p* < 0.05).

### 3.3. Gene Expression

#### 3.3.1. Genes Related to Antioxidant Response

**Figure 4:**
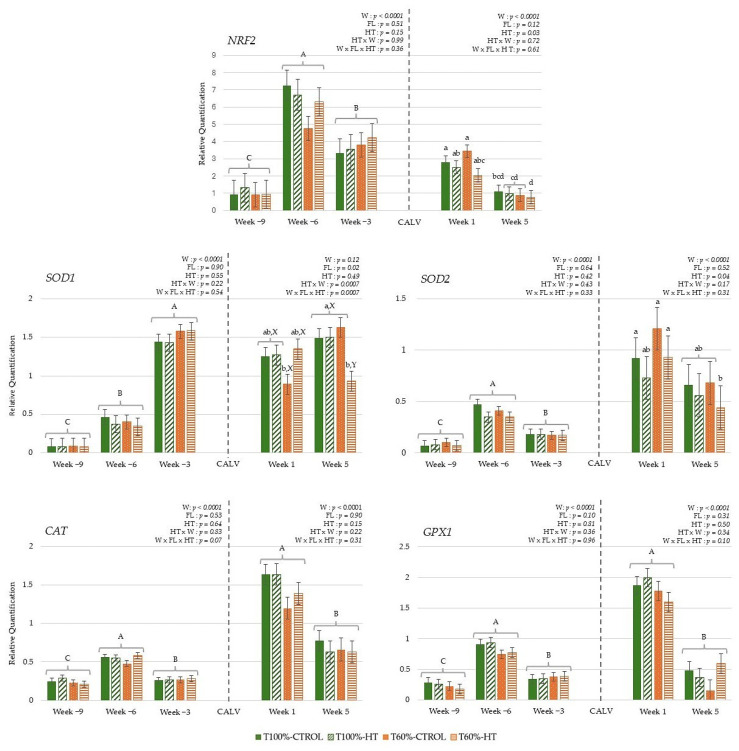
Peripheral gene expression related to antioxidant status (nuclear factor erythroid 2-related factor 2 (*NRF2*), superoxide dismutase 1 (*SOD1*), superoxide dismutase 2 (*SOD2*), catalase (*CAT*), glutathione peroxidase 1 (*GPX1*)), according to week (W) relative to calving (CALV), feeding level (FL), and hydroxytyrosol (HT) supplementation in cows, during gestation (T100%-CTROL, *n* = 11; T100%-HT, *n* = 10; T60%-CTROL, *n* = 14; T60%-HT, *n* = 11) and during lactation (T100%-CTROL, *n* = 10; T100%-HT, *n* = 9; T60%-CTROL, *n* = 9; T60%-HT, *n* = 9). Means with different letters differ at *p* < 0.05. If only the week has a significant effect, its letters (A, B, C) are shown. If there are any other significant effects, letters from the triple interaction (a, b, c, d) are used, and if any interactions between feeding level or supplementation with week occur, differences within the same week (X, Y) are also included. If no significant effects are detected, no letters are shown. Groups represented with the same letters between gestation and lactation are not related.

The results during gestation did not reveal a significant effect of undernutrition or HT supplementation, but the week affected all genes (Figure 4). In general, antioxidant defences, namely, *NRF2*, *SOD2*, *CAT*, and *GPX1*, showed the lowest expression at week −9 and peaked at week −6, followed by a decrease at week −3 (*p* < 0.0001). However, *SOD1* expression increased linearly as gestation progressed to week −3 (*p* < 0.05). After calving, there were differences in the interaction among week, feeding level, and HT supplementation prepartum in *SOD1* expression, which was the lowest in the T60%-HT cows compared with the rest of the dietary treatments (*p* < 0.05). Meanwhile, there was lower *NRF2* and *SOD2* expression in the HT cows compared to the CTROL cows during the lactation period (*p* < 0.05). Regarding the week effect during the lactation period, antioxidant defences (*NRF2*, *SOD2*, *CAT*, and *GPX1*) decreased significantly in expression at week 5 compared to week 1 (*p* < 0.0001).

**Figure 5:**
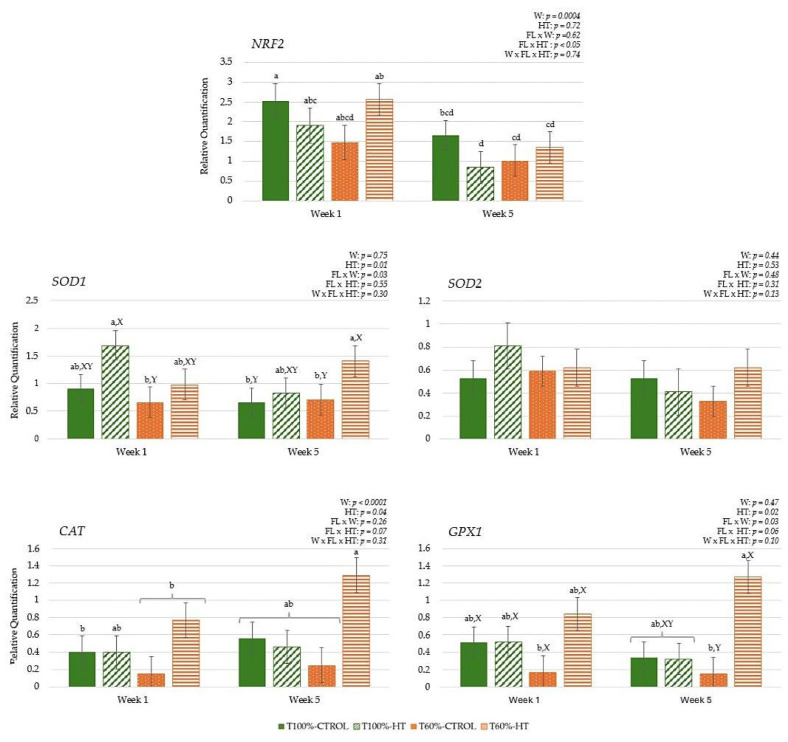
Interaction between maternal feeding level (FL) and hydroxytyrosol (HT) supplementation on peripheral gene expression related to antioxidant status (nuclear factor erythroid 2-related factor 2 (*NRF2*), superoxide dismutase 1 (*SOD1*), superoxide dismutase 2 (*SOD2*), catalase (*CAT*), glutathione peroxidase 1 (*GPX1*)), in calves at given weeks (W) relative to birth (T100%-CTROL, *n* = 10; T100%-HT, *n* = 9; T60%-CTROL, *n* = 9; T60%-HT, *n* = 9). Means with different letters differ at *p* < 0.05. If there are any other significant effects, letters from the triple interaction (a, b, c, d) are used. If interactions between maternal treatment and week occur, differences within the same week (X, Y) are also included. If no significant effects are detected, no letters are shown.

In the calves, antioxidant genes were greater in the HT-supplemented and higher-fed groups during the first week, with some antioxidant responses decreasing and others increasing by week 5, indicating that both diet and age influenced oxidative status (Figure 5). The interaction of feeding level with HT supplementation significantly affected transcription factor *NRF2*, with high expression levels observed in the T100%-CTROL and T60%-HT groups compared with the T100%-HT and T60%-CTROL groups (*p* < 0.05). The interaction of feeding level per week of age affected *SOD1* expression, which showed greater expression in the T100%-HT group than the T60%-CTROL group at week 1 and greater expression in the T60%-HT group compared to the T60%-CTROL at week 5 (*p* < 0.05). Accordingly, greater *GPX1* expression levels were observed in the T60%-HT group compared to the T60%-CTROL group at week 5 (*p* < 0.05). Meanwhile, the gene expression of antioxidant enzymes (*SOD1*, *CAT*, and *GPX1*) was greater in the HT cows compared with the CTROL groups, regardless of the feeding level (*p* < 0.05). The expression of *NRF2* was decreased at week 5 as compared to week 1, contrary to *CAT*, which showed an increase at week 5 (*p* < 0.05).

#### 3.3.2. Genes Related to Immune Response

**Figure 6:**
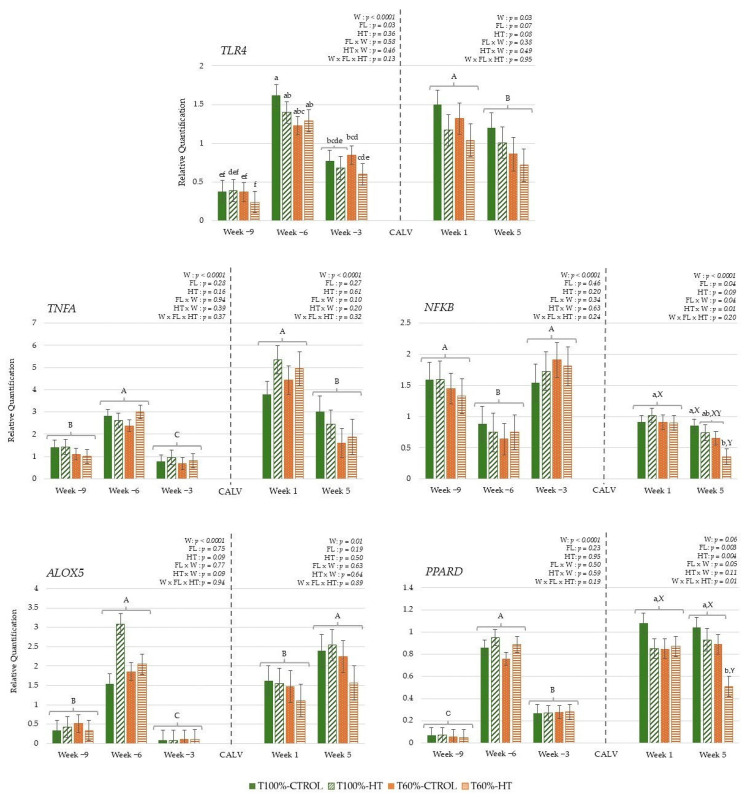
Peripheral gene expression related to immune response (toll-like receptor 4 (*TLR4*), tumor necrosis factor alpha (*TNFA*), nuclear factor kappa B (*NFKB*), 5-lipoxygenase (*ALOX5*), peroxisome proliferator-activated receptor delta (*PPARD*)), according to week (W) relative to calving (CALV), feeding level (FL), and hydroxytyrosol (HT) supplementation in cows, during gestation (T100%-CTROL, *n* = 11; T100%-HT, *n* = 10; T60%-CTROL, *n* = 14; T60%-HT, *n* = 11) and during lactation (T100%-CTROL, *n* = 10; T100%-HT, *n* = 9; T60%-CTROL, *n* = 9; T60%-HT, *n* = 9). Means with different letters differ at *p* < 0.05. If only the week has a significant effect, its letters (A, B, C) are shown. If there are any other significant effects, letters from the triple interaction (a, b, c, d, e, f) are used, and if any interactions between feeding level or supplementation with week occur, differences within the same week (X, Y) are also included. If no significant effects are detected, no letters are shown. Groups represented with the same letters between gestation and lactation are not related.

The results during gestation did not reveal differences based on HT supplementation, but they did reveal differences based on feeding level; lower overall *TLR4* expression was observed in the T60% cows compared to the T100% cows during gestation (*p* < 0.05). Regarding the week effect, immune genes, namely, *TLR4*, *PPARD*, *TNFA*, and *ALOX5*, showed lower expression at week −9 relative to calving and increased subsequently at week −6, followed by a decrease at week −3. In contrast, transcription factor *NFKB* gene expression decreased at week −6 and then increased at week −3, reaching similar levels to those observed at week −9 (Figure 6). During lactation, the gene expression of transcription factors *PPARD* and *NFKB* was affected by the interaction among week postpartum, prepartum feeding level, and HT supplementation. At week 5, the T60%-HT group exhibited the lowest *PPARD* expression levels among all cow groups, and these cows also showed significantly lower *NFKB* expression compared with the T100%-CTRL group (*p* < 0.05). Some genes showed differences in their expression based on the week effect, with decreased *TLR4* and *TNFA* expression at week 5 compared to week 1, but increased *ALOX5* expression at week 5 compared to week 1 (*p* < 0.05) (Figure 6).

**Figure 7:**
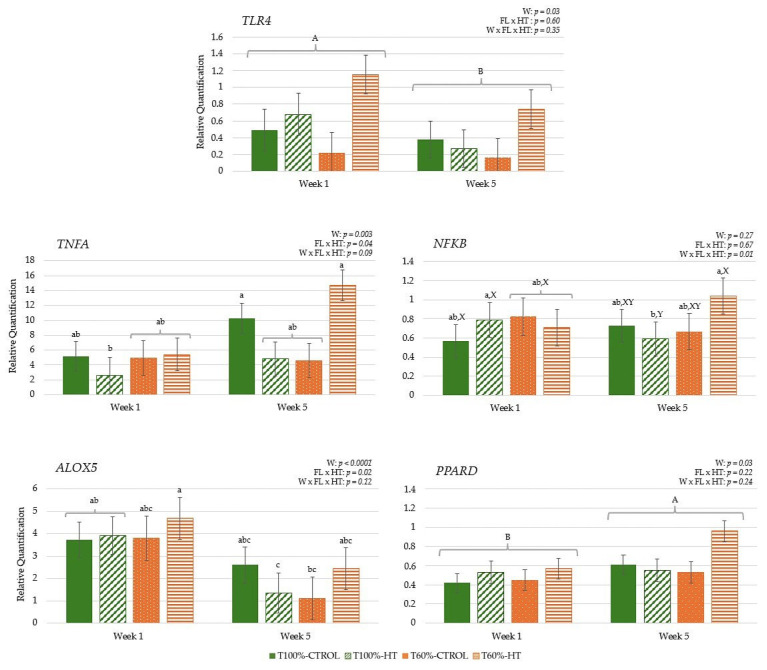
Interaction between maternal feeding level (FL) and hydroxytyrosol (HT) supplementation on peripheral gene expression related to immune response (toll-like receptor 4 (*TLR4*), tumor necrosis factor alpha (*TNFA*), nuclear factor kappa B (*NFKB*), 5-lipoxygenase (*ALOX5*), peroxisome proliferator-activated receptor delta (*PPARD*)), in calves at given weeks (W) relative to birth (T100%-CTROL, *n* = 10; T100%-HT, *n* = 9; T60%-CTROL, *n* = 9; T60%-HT, *n* = 9). Means with different letters differ at *p* < 0.05. If only the week has a significant effect, its letters (A, B) are shown. If there are any other significant effects, letters from the triple interaction (a, b, c) are used. If interactions between maternal treatment and week occur, differences within the same week (X, Y) are also included. If no significant effects are detected, no letters are shown.

In the calves, pro-inflammatory immune-related pathways were influenced by the interaction between week, feeding level, and HT supplementation, with early increases in pro-inflammatory genes followed by later changes in regulatory pathways, reflecting dynamic diet- and time-dependent effects. At week 5, *NFKB* expression levels were significantly lower in the T100%-HT group compared to the T100%-HT group at week 1 and the T60%-HT group at week 5 (*p* < 0.05) (Figure 7). The expression of pro-inflammatory immune genes *TNFA* and *ALOX5* was also significantly affected by the interaction between feeding level and supplementation, with greater *TNFA* and *ALOX5* expression observed in the T100%-CTROL and T60%-HT groups (*p* < 0.05). *TLR4* and *ALOX5* expression was significantly decreased at week 5; meanwhile, the expression of *TNFA* and *PPARD* was significantly increased at week 5 as compared to week 1 (*p* < 0.05).

#### 3.3.3. Genes Related to Energy Metabolism

**Figure 8:**
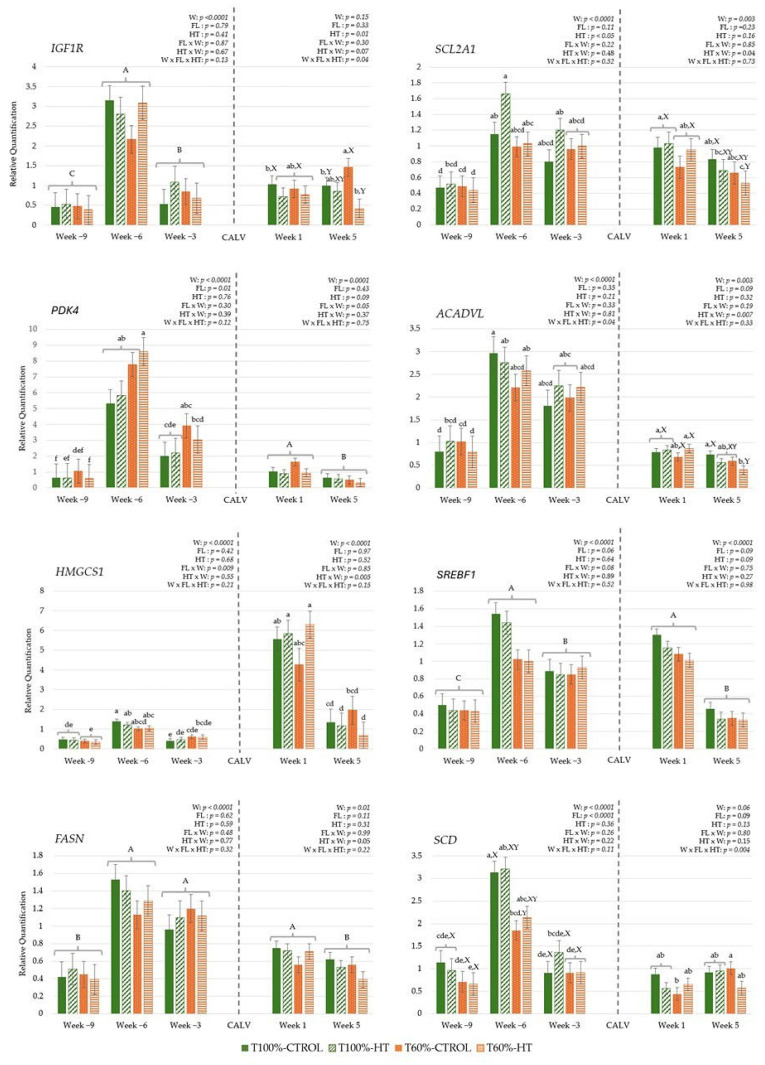
Peripheral gene expression related to energy metabolism (insulin-like growth factor 1 receptor (*IGF1R*), the gene encoding solute carrier family 2 member 1 (*SLC2A1*/*GLUT1*), pyruvate dehydrogenase kinase isoenzyme 4 (*PDK4*), acyl-CoA dehydrogenase (*ACADVL*), 3-hydroxy-3-methylglutaryl-CoA synthase 1 (*HMGCS1*), sterol regulatory element-binding protein 1 (*SREBF1*), fatty acid synthase (*FASN*), stearoyl-CoA desaturase (*SCD*)), according to week (W) relative to calving (CALV), feeding level (FL), and hydroxytyrosol (HT) supplementation in cows, during gestation (T100%-CTROL, *n* = 11; T100%-HT, *n* = 10; T60%-CTROL, *n* = 14; T60%-HT, *n* = 11) and during lactation (T100%-CTROL, *n* = 10; T100%-HT, *n* = 9; T60%-CTROL, *n* = 9; T60%-HT, *n* = 9). Means with different letters differ at *p* < 0.05. If only the week has a significant effect, its letters (A, B, C) are shown. If there are any other significant effects, letters from the triple interaction (a, b, c, d, e, f) are used, and if any interactions between feeding level or supplementation with week occur, differences within the same week (X, Y) are also included. If no significant effects are detected, no letters are shown. Groups represented with the same letters between gestation and lactation are not related.

The interaction between week, feeding level, and HT supplementation during gestation showed that the long-chain fatty acid oxidation key enzyme *ACADVL* increased less in the T60%-CTROL group compared to the rest of the cow groups from week −9 to −6 (Figure 8). Moreover, at week −6, the expression levels in the T60%-HT group were statistically equal to those in the T100%-CTROL group (*p* > 0.05). Regarding feeding level, significantly greater gene expression of the cholesterol biosynthesis enzyme *HMGCS1* was observed in the T100% cows at week −6 (*p* < 0.05) compared to the T100% and T60% cows at week −9 and week −3. Additionally, greater gluconeogenic (*PDK4*) but lower fatty acid desaturation enzyme (*SCD*) pathways were observed in the T60% cows compared to the T100%-cows during gestation (*p* < 0.05). Regarding HT supplementation, glucose transporter, *SLC2A1*/*GLUT1*, gene expression was greater in the HT cows compared to the CTROL cows (*p* < 0.05). *SLC2A1*/*GLUT1* and fatty acid synthase enzyme *FASN* gene expression also increased from week −9 to week −6 and then remained constant until week −3. Likewise, IGF signalling (*IGF1R*), gluconeogenic (*PDK4*) and lipid synthesis pathways (*SCD*, *SREBF1*) showed increased expression levels up to week −6, followed by a decrease in week −3 (*p* < 0.0001).

During lactation, the interaction among week, feeding level, and HT supplementation revealed greater *IGF1R* expression in the cows from the T60%-CTROL group at week 5 compared to the cows from the T100%-CTROL and T60%-HT groups. Meanwhile, higher *SCD* expression was observed in the T60%-CTROL cows at week 5 compared to the T60%-CTROL cows at week 1 (*p* < 0.05). Regarding HT supplementation per week, there was greater glucose transporter (*SLC2A1*/*GLUT1*), long-chain fatty acid oxidation (*ACADVL*) and lipid biosynthesis (*HMGCS1*) gene expression in the HT cows at week 1 compared to the CTROL cows at the same week, but there was lower expression in the HT cows at week 5 compared to the CTROL cows at week 5 (*p* < 0.05). Some genes also showed differences in their expression based on the week effect, with significantly decreased *PDK4*, *SREBF1*, and *FASN* expression at week 5 compared to week 1 (*p* < 0.05).

**Figure 9:**
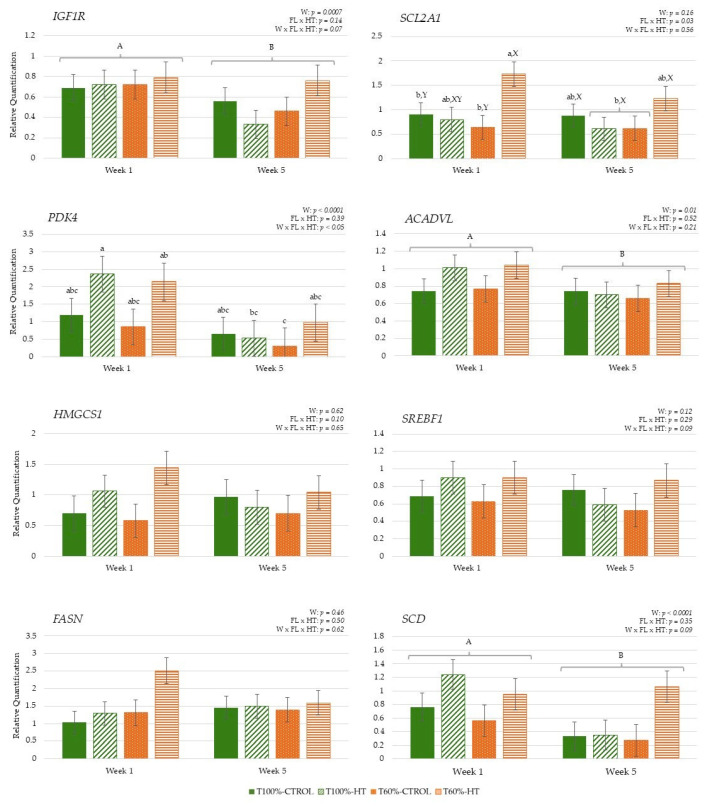
Interaction between maternal feeding level (FL) and hydroxytyrosol (HT) supplementation on peripheral gene expression related to energy metabolism (insulin-like growth factor 1 receptor (*IGF1R*), the gene encoding solute carrier family 2 member 1 (*SLC2A1*/*GLUT1*), pyruvate dehydrogenase kinase isoenzyme 4 (*PDK4*), acyl-CoA dehydrogenase (*ACADVL*), 3-hydroxy-3-methylglutaryl-CoA synthase 1 (*HMGCS1*), sterol regulatory element-binding protein 1 (*SREBF1*), fatty acid synthase (*FASN*), stearoyl-CoA desaturase (*SCD*)), in calves at given weeks (W) relative to birth (T100%-CTROL, *n* = 10; T100%-HT, *n* = 9; T60%-CTROL, *n* = 9; T60%-HT, *n* = 9). Means with different letters differ at *p* < 0.05. If only the week has a significant effect, its letters (A, B) are shown. If there are any other significant effects, letters from the triple interaction (a, b, c) are used. If interactions between maternal treatment and week occur, differences within the same week (X, Y) are also included. If no significant effects are detected, no letters are shown.

In the calves, maternal HT supplementation and feeding level influenced early metabolic activity in blood cells, enhancing glucose metabolism in the first week, while lipid-related pathways decreased over time, reflecting diet- and time-dependent effects. The interaction between feeding level and HT supplementation across weeks showed greater *PDK4* expression in T100%-HT and T60%-HT at week 1 compared to the T60%-CTROL group at week 5 (*p* < 0.05). On the other hand, the interaction between feeding level at supplementation was significantly affected in T60%-HT, with greater glucose transport *SCL2A1*/*GLUT1* expression compared to T60%-CTROL and T100%-CTROL, mainly at week 1 (*p* < 0.05). The expression of *IGF1R*, long-chain fatty acid oxidation enzyme (*ACADVL*), and fatty acid desaturation enzyme (*SCD*) decreased at week 5 as compared to week 1 (*p* < 0.05) (Figure 9).

## 4. Discussion

This study was designed to characterize the effects of undernutrition and HT supplementation during the last third of gestation on oxidative status, immune regulation, and metabolic pathways in cows during late pregnancy. It also assessed the effects in cows and their calves just after parturition (week 1) and in the early postpartum period (week 5). Our main findings were that in pregnant cows, undernutrition favoured gluconeogenic pathways (*PDK4*) and decreased lipid synthesis (*HMGCS1* and *SCD*) and TLR signalling. This suggests an enhanced catabolic/tolerogenic function in blood cells in response to dietary nutrient restriction in late pregnancy. After parturition, T60% cows still sustained *SCD* inhibition of saturated fatty acids, with diminished antioxidant *SOD1* expression in blood cells. However, a recovery was observed in the early postpartum period, with increased lipid desaturation and IGF signalling. HT supplementation increased glucose transport (*SLC2A1*/*GLUT1*) during late pregnancy. After parturition (week 1), prenatal HT supplementation increased glucose transport (*SLC2A1*/*GLUT1*) and cholesterol synthesis (*HMGCS1*) and blunted antioxidant responses (*NRF2* and *SOD2*) in cows but increased antioxidant defences (*SOD1*, *CAT*) in calves. Interestingly, HT supplementation caused different effects depending on maternal metabolic status. In T60%-cows, HT increased long-chain fatty acid beta-oxidation (*ACADVL*) at week −6 and after parturition, while early postpartum was characterized by diminished *ACADVL*, IGF signalling (*IGF1R*), immune regulation (*NFKB*, *PPARD*), and antioxidant responses (*SOD1*) and improved antioxidant capacity (increased ABTS). In calves from T60%-group, HT supplementation increased glucose transport (*SLC2A1*/*GLUT1*) and gluconeogenic pathways (*PDK4*), which pointed to enhanced immune responses (*TNFA*, *ALOX5* (at week 1), and *NFKB* (week 5)) and antioxidant capacity (ABTS, *GPX1* (week 1), and *NRF2* (week 5)) during their first month of life.

Late pregnancy is marked by endocrine adjustments that prepare the cow for parturition and lactogenesis [49]. Both milk production and immune defences require reprograming to ensure glucose availability to the respective cell types with increased rates of lipid mobilization and decreased rates of lipid synthesis pathways in adipose tissue. During this period, leukocyte function must undergo an intracellular metabolic shift from oxidative phosphorylation, associated with anti-inflammatory responses during pregnancy, to mainly aerobic glycolysis, associated with a pro-inflammatory response during parturition [50,51]. Late pregnancy (week −6) was characterized by a marked immunoactivation with an increase in energy supply, with the transcription of major regulators of lipid metabolism, IGF signalling, gluconeogenic and glycolytic genes, and antioxidant defences that seemed to support the requirements of a pro-inflammatory response. However, an altered immune response before parturition [52] caused a slight decrease in immune function and glycolytic pathways for energy supply at the end of the study period (week −3). These immunometabolic patterns were associated with changes in the somatotropic axis and glucocorticoid response. The ‘uncoupling of the somatotropic axis’ reduces insulin sensitivity in peripheral tissues and favours the supply of glucose to lactocytes [53]. Thus, circulating immune cells rely on the rate of GLUT expression during activation to maintain glucose disposal and shift from insulin-dependent (*GLUT4*) to insulin-independent glucose transporter expression (*GLUT1*/*GLUT3*) [54]. Circulating blood cells probably rely on *SLC2A1*/*GLUT1* expression since it increased in week −6 and was maintained during the study period. In this trial, undernutrition in the last third of pregnancy caused fat mobilization in dams, which was reflected in higher plasma NEFA and cholesterol, and lower IGF-1, glucose, and fructosamine in T60% than T100% cows. These energy metabolism responses prioritized calf fetus growth, since calf body weight at birth was similar, regardless of maternal feeding level [29]. Direct contact between cells and circulating metabolites during this period influenced gene expression in circulating leukocytes during late pregnancy. Accordingly, maternal undernutrition promoted higher expression of *PDK4* during pregnancy in T60% cows, a key gene in gluconeogenesis that encodes a kinase that inhibits pyruvate metabolism in the TCA cycle and favours fatty acid oxidation. In addition, decreased expression rates of pathways stimulating lipid synthesis and TLR signalling were observed in T60% dams. Increased fatty acid synthesis is necessary for proper immune cell differentiation during TLR activation to facilitate signalling in response to external stimuli [55,56]. *TLR4* is mainly expressed in neutrophils and macrophages, playing a key role in the activation of the inflammatory response, associated with the expression of *TNFA*, a pro-inflammatory cytokine involved in the regulation of inflammation, immune cell recruitment, and apoptosis [57]. Recent studies have shown that the type of fatty acid being synthesized governs the type of cytokines being made by macrophages [56] and T helper cells [58]. Thus, overfeeding leads to increased inflammatory activity in various species, as adipose tissue is pro-inflammatory by nature, which results in greater activation of TLR4 [59,60]. Hence, the inhibition of TLR4 signalling is likely to be related to an increase in oxidative phosphorylation and consumption of fatty acids in mitochondria, related to an anti-inflammatory phenotype. Consequently, maternal undernutrition during pregnancy enhanced metabolism to catabolic pathways and decreased genes involved in lipid synthesis in blood leukocytes, with decreased expression of *HMGCS1*, the cytosolic enzyme 3-hydroxy-3-methylglutaryl-coenzyme A synthase 1 involved in endogenous cholesterol biosynthesis [61], and decreased *SCD* expression. Decreased *SCD* expression could be related to lower activity of its upstream regulators, such as sterol regulatory element-binding protein (SREBP). However, in the present study, no significant differences were observed in the expression of this gene. The observed immunometabolic pathways in response to diet restriction are consistent with previous studies performed in ruminants [5,19,62].

After parturition, dams experienced physiological inflammation linked to the inherent stress of parturition [51,63], with a marked increase in pro-inflammatory response (*TLR4*, *TNFA*, *NFKB*) and enhanced metabolism of lipid biosynthesis (*SREBF1*, *HMGCS1*, *FASN*), long-chain fatty acid oxidation (*ACADVL*) and gluconeogenic (*PDK4*) pathways, and antioxidant defences (*NRF2*, *SOD2*, *CAT* and *GPX1*) in blood cells. In oxidative stress conditions, Nrf2, involved in mitochondrial biogenesis, regulates antioxidant enzymes such as SOD2, which catalyzes the dismutation of superoxide into oxygen and H_2_O_2_ within mitochondria; CAT, which degrades H_2_O_2_ into water and oxygen; and GPX1, which reduces lipid hydroperoxides to alcohol and also converts H_2_O_2_ into water [64,65]. Oxidative stress is generally believed to be a mediator of insulin resistance, and it can be associated with a slight increase in MDA levels, a stable end-product of lipid peroxidation, before and after parturition, both in cows and calves, and with an increase in ROS production and NEFA mobilization [66]. ROS are continuously produced by normal metabolic processes, but the production rate might increase significantly under conditions of high metabolic demand [67]. At the beginning of the last third of gestation (week −9), MDA levels were influenced by feeding level, with undernourished cows having the highest values [68,69,70]. This suggests a great mobilization of body fat tissues, at the cost of, among other factors, more weight and body condition loss during this period and later, such as early lactation, to meet the high metabolic demands [29], reaching optimal or normal MDA levels before parturition [71,72,73]. As lactation progressed, all dams exhibited an increase in MDA but also in antioxidant capacity. In general, an immunometabolic pro-inflammatory phenotype decreased, but an increase in *ALOX5* was observed, which encodes the enzyme 5-lipoxygenase and participates in the biosynthesis of leukotrienes, in addition to being an indicator of leukocyte maturation in cows [74]. After parturition (week 1), when both lactation and immune response impose high glucose demands, T60% cows still sustained catabolic metabolism in blood cells with diminished fatty acid desaturation (*SCD*) and lower *SOD1* expression, which converts the reactive superoxide radical into H_2_O_2_ within the cytoplasmic environment, probably associated with a lower immune–redox response. Subsequent metabolic recovery and probably cell activation capacity were observed in early postpartum (week 5) with increased fatty acid desaturation and IGF signalling in these dams. These patterns reflect a shift from catabolic to anabolic pathways; however, these changes can induce immunosuppressive responses and inflammatory states that affect the immune system’s ability to respond effectively to infections, often without clinical manifestation [75,76].

The benefits of olive polyphenol supplementation in mitigating oxidative and immunometabolism stress caused by undernutrition and the physiological demands of pregnancy were confirmed in early lactation. A large body of published work has demonstrated the antioxidant and anti-inflammatory properties of HT and olive oil [77]. However, the redox reactions produced in living cells following HT supplementation are poorly understood. Although there was no antioxidant effect during pregnancy, possibly due to the immunosuppressive effect of the progesterone environment in pregnant dams [78], HT diminished *NRF2* and *SOD2* gene expression after calving, probably thanks to its ability to scavenge free radicals. HT has been demonstrated to reduce ROS generation by promoting mitophagy and Nrf2 modulation [79,80]. The Kelch-like ECH-associated protein 1 (Keap1)/nuclear factor erythroid 2-related factor 2 (Nrf2)/antioxidant response element (ARE) signalling pathway serves as a crucial endogenous cellular antioxidant system, regulating the expression of downstream antioxidant enzymes to alleviate oxidative stress-induced damage, and provides a mechanistic context for the observed modulation of antioxidant defences by HT [81]. In murine models, HT also alters the molecular regulators of glucose uptake through epigenetic mechanisms or redox reactions [82,83]. In this study, HT supplementation promoted insulin-independent glucose uptake in blood cells, with increased gene expression of glucose transport (*SLC2A1*/*GLUT1*) during late pregnancy and just after parturition (week 1). During periods of oxidative stress, plasma glucose levels tend to increase, as an adaptation mechanism, leading to a decrease in *SLC2A1*/*GLUT1* expression in some animal species [84,85]. According to our results, *SLC2A1*/*GLUT1* expression was not affected by diet during the peripartum period, as observed in monocytes in peripartal dairy cows with different dietary energy supplies [54]. Hence, HT would help to mitigate oxidative stress and probably stabilize cell glucose levels, leading to the positive regulation of this gene. Interestingly, prepartum HT supplementation affected the peripheral blood immunometabolic and redox pathways differently depending on the feeding treatment and the period relative to calving. In T60%-cows, with decreased lipid synthesis, HT increased long-chain fatty acid beta-oxidation (*ACADVL*) at week −6 and just after parturition (week 1), highlighting a compensatory mechanism in response to maternal undernutrition [61,86] that was not observed in T100%-HT cows. Metabolic rewiring towards enhanced long-chain fatty acid oxidation seems to support not only the anti-inflammatory phenotype in macrophages and regulatory T cell formation, but also pro-inflammatory functions in monocytes during glucose deprivation in in vitro models [87]. Accordingly, a moderate increase in NEFA and cholesterol levels and a decrease in IGF-1 levels were observed in T60%-HT [29]. In early postpartum (week 5), T60%-HT still sustained an improved total antioxidant capacity with respect to the week after parturition. However, in this period, when most of the total body glucose is directed to the mammary gland, a decrease in *SLC2A1*/*GLUT1* expression was observed that entailed diminished pro-inflammatory immune regulation (*NFKB*, *PPARD*), long-chain fatty acid oxidation (*ACADVL*), IGF signalling (*IGF1R*), and antioxidant responses (*SOD1*). This response was not observed, however, in T60%-CTROL dams. Eger et al. [54] reported that the monocyte glucose transporter was negatively correlated with lactose yield and, therefore, with milk production, in accordance with our results, as the peak of lactogenesis in beef cows occurs later than the conclusion of our study, at 6 to 8 weeks postpartum [88]. These results point to a better immune status in HT-supplemented dams that corresponded with higher plasma IgG concentrations, and higher colostrum IgM and IgG concentrations [29].

The improved maternal metabolic status in HT-supplemented dams was reflected in calves that were heavier at birth [29]. The first weeks of life of calves constitute a critical and highly dynamic period from a metabolic standpoint, requiring its own homeostatic regulation. Young calves exhibit bioenergetic pathways that involve both mitochondrial respiration and glycolysis [89]. Accordingly, the first week of life of calves was characterized by activation of pro-inflammatory pathways (*TLR4* and *ALOX5*) and fatty acid biosynthesis pathways (*SCD*), which could promote cell membrane formation [63], supported by IGF signalling [90,91], long-chain fatty acid oxidation (*ACADVL*), and stabilizing pyruvate pathways (*PDK4*). This indicates the preservation of glucose oxidation, favouring gluconeogenesis and the need to obtain energy through oxidation of very long-chain fatty acids [92,93,94]. Higher expression of *NRF2*, a signal of an enhanced antioxidant response and mitochondrial biosynthesis, was also recorded as a response to the oxidative stress generated at birth and the rapid growth and immune activation of the calf [16,64]. In five-week-old calves, higher expressions of *TNFA* and *PPARD* were observed in the progeny from T60%-HT dams, suggesting an enhancement in mitochondrial function and cellular defence systems [95]. In addition to this, there was an increase in the expression of *CAT*, probably counteracting the increase in lipid peroxidation (higher MDA levels) [64]. In this period, glucose use is initially prioritized in other tissues, followed by a subsequent progression towards controlled inflammation, regulation, and maturation of the immune response, antioxidant system, and metabolism [17,96].

The benefits of maternal olive polyphenol supplementation, depending on maternal dietary treatment, were also confirmed in calves. Our results suggest that maternal undernutrition, without HT supplementation, could have rewired calf immunometabolism towards catabolic and less inflammatory pathways [94,97]. Accordingly, changes in calf endocrine regulation were observed as elevated plasma cortisol levels in newborn calves from T60% dams [29]. HT supplementation during the last third of gestation had a favourable modulatory effect on T60% calves, with higher expression of *TNFA*, *TLR4*, and *ALOX5*, in comparison with the levels reached by T100%-CTROL. Metabolically, total antioxidant capacity, glucose transport (*SLC2A1*/*GLUT1*), especially for T60%-HT calves compared to T60%-CTROL calves, and gluconeogenic pathways were observed in calves born from mothers receiving HT. Similar results have been observed with other types of antioxidants in various tissues [98,99,100]. HT supplementation caused a similar high expression of *NRF2* in T60%-HT and T100%-CTROL and a high expression of *CAT* in calves whose mothers received HT, regardless of the feeding level, suggesting an improved redox environment in blood cells [101,102]. In summary, these results point to better antioxidant regulation, more stable insulin levels, and better glucose uptake and transport, reinforcing the growth and development of T60% calves [98,103]. However, this was not the case for the T100% group, where lessened regulation of these markers was observed during the first week [85], and others, such as *NRF2*, *TNFA*, and *ALOX5*, throughout the study period. It is likely that the availability of exogenous antioxidants obtained through colostrum reduced the need to activate pathways involved in the endogenous production of antioxidants [104,105,106]. In five-week-old calves, modulation of maternal HT supplementation still affected T60% calves, with higher levels of *NFKB*, unlike T100% calves, who showed a decrease in this gene at week 5. Additionally, T60%-HT calves also showed higher expression levels of antioxidant enzymes *SOD1* and *GPX1* at week 5 compared to T60%-CTROL calves, suggesting more intense activation of the antioxidant system, as an adaptive measure against oxidative stress and immunometabolic response [72,73,102].

This study was limited by its relatively short postpartum follow-up (5 weeks), moderate sample size, and lack of functional immune or health performance outcomes in calves. Future studies should explore whether the immunometabolic modulation observed here translates into improved long-term growth or lifespan disease resistance.

## 5. Conclusions

In conclusion, nutrient restriction during the last third of pregnancy caused immunometabolic adaptations towards catabolic pathways in peripheral blood, with limited long-term effects after parturition. Dietary HT promoted a higher gene expression of glucose transport, metabolic regulation, and long-chain fatty acid beta-oxidation, highlighting a compensatory mechanism in response to maternal undernutrition in nutrient-restricted dams and their calves. Interestingly, dietary HT also improved plasma antioxidant activity and modulated the key genes involved in pro-inflammatory immune responses in the offspring. Our findings may contribute to identifying the potential benefits of olive polyphenol supplementation in mitigating oxidative and immunometabolism stress caused by undernutrition and the physiological demands of pregnancy and early lactation. These results suggest that HT may be a useful additive in low-input beef systems to improve offspring resilience under maternal nutritional stress.

## Figures and Tables

**Table 1:** Primer sequences and sources.

Gene	General Gene Role	Forward and Reverse Primer (5′–3′)	Base Pair	Access. No.	Efficiency (%)	nM	Source
*RPL19*	Housekeeping	F: GATCCGGAAGCTGATCAAAG R: ATTCGAGCATTGGCAGTACC	147	NM_001040516.1	97.5	200	[38]
*ACTB*	Housekeeping	F: CTGGACTTCGAGCAGGAGAT R: GATGTCGACGTCACACTTC	207	AY141970	105	200	[39]
*SOD1*	Antioxidant enzyme	F: CACCATCCACTTCGAGGCAA R: GCACTGGTACAGCCTTGTGT	126	NM_174615.2	95.1	300	[40]
*SOD2*	Antioxidant enzyme	F: GGATCCCCTGCAAGGAACAA R: TGGCCTTCAGATAATCGGGC	110	NM_201527.2	98.6	300	[40]
*CAT*	Antioxidant enzyme	F: TCACTCAGGTGCGGACTTTC R: GGATGCGGGAGCCATATTCA	162	NM_001035386.2	111.1	300	[40]
*GPX1*	Antioxidant enzyme	F: GAGCCCTTCAACCTGTCCTC R: GCGTTTTCCTGATGCCCAAAC	179	NM_174076.3	95.5	250	[41]
*NRF2*	Transcription factor	F: AGCTCAGCATGATGGACTTGGA R: CAGCTCATGCTCCTTCTGTCG	152	NM_001011678.2	101.1	333	[41]
*TLR4*	Immune response	F: TCCCCGACAACATCCCCATA R: AAAGGCTCCCCAGGCTAAAC	224	NM_174198	103.2	125	[42]
*NFKB*	Transcription factor	F: CGGGGACTACGACCTGAATG R: GCCTGGTCCCGTGAAATACA	250	NM_001080242	95.5	250	[42]
*TNFA*	Immune response	F: CCAGAGGGAAGAGCAGTCC R: GGCTACAACGTGGGCTACC	112	NM_173966.3	89.9	125	[42]
*ALOX5*	Inflammation regulation	F: GCAGGAAGACCGCATGTTTG R: GTTCCCTTGCTCGATCTCCT	163	NM_001192792	107.2	200	[43]
*PPARD*	Transcription factor	F: TGTGGCAGCCTCAATATGGA R: GACGGAAGAAGCCCTTGCA	100	NM_001083636.1	106.2	400	[44]
*PDK4*	Energy metabolism	F: TGTATCCCAAGCAAGGAACC R: TTTGATCCCTTAGCGTGTCC	86	NM_001101883.1	99.9	400	[5]
*SCD*	Energy metabolism	F: CAGCGGAAGGTCCCGA R: CAAGTGGGCCGGCATC	157	NM_173959.4	90.5	400	[45]
*IGF1R*	Energy metabolism	F: TTAAAATGGCCAGAACCTGAG R: ATTATAACCAAGCCTCCCAC	314	NM_001244612.1	104	400	[46]
*SREBF1*	Energy metabolism	F: CCAGCTGACAGCTCCATTGA R: TGCGCGCCACAAGGA	67	NM_001113302	95.7	400	[47]
*FASN*	Energy metabolism	F: CTGAGTCGGAGAACCTGGAG R: ACAATGGCCTCGTAGGTGAC	232	NM_001012669	90.3	400	[47]
*HMGCS1*	Energy metabolism	F: TGTACGGCTCCCTGGCTTCTG R: CATGTTCCTTCGAAGAGGGAATC	313	BC_102850	93.5	200	[47]
*SLC2A1*/*GLUT1*	Energy metabolism	F: GCTTCTCCAACTGGACTTCG R: ACAGCTCCTCAGGTGTCTTG	225	NM_174602	99.8	250	[48]
*ACADVL*	Energy metabolism	F: TCCCCAAACTGGCATCTGGG R: ATGGGTGACGCCGCCAAAGC	275	BC_103104	93.2	400	[47]

**Table 2:** Maternal feeding level (FL) and hydroxytyrosol (HT) supplementation effects on cow performances and circulating HT metabolite uptake to serum and colostrum ^1^.

					*p*-Values		
	T100%-CTROL	T100%-HT	T60%-CTROL	T60%-HT	FL	HT	FL × HT
Gestation (*n*)	11	10	14	11			
BW at −12 weeks before calving (kg)	672 ± 15.3	683 ± 16.4	669 ± 13.5	668 ± 15.3	0.56	0.76	0.69
ADG ^2^ during 12 weeks prepartum (kg/day)	0.62 ± 0.08 ^a^	0.43 ± 0.08 ^a^	0.03 ± 0.07 ^b^	0.15 ± 0.08 ^b^	<0.001	0.66	0.05
BW after calving (kg)	679 ± 6.4 ^a^	666 ± 6.5 ^a^	625 ± 5.3 ^b^	624 ± 6.2 ^b^	<0.001	0.24	0.34
Postpartum (*n*)	10	9	9	9			
ADG (5 weeks postpartum (kg/day))	−0.71 ± 0.23 ^a^	−0.66 ± 0.24 ^a^	−0.06 ± 0.25 ^b^	−0.30 ± 0.25 ^b^	0.04	0.69	0.56
Cows with key HT metabolites detected in serum at week −3 before calving ^3^ (*n*)	6	6	6	6			
Hydroxytyrosol sulphate (HTS)	0/6 ^a^	5/6 ^b^	0/6 ^a^	5/6 ^b^	1.00	<0.001	1.00
Alcohol homovanillic sulphate (AHVS)	0/6 ^a^	5/6 ^b^	0/6 ^a^	5/6 ^b^	1.00	<0.001	1.00
Both AHVS and HTS	0/6 ^a^	4/6 ^b^	0/6 ^a^	4/6 ^b^	1.00	<0.001	1.00
Cows with key HT metabolites detected in colostrum (<12 h postpartum) (*n*)	6	6	6	6			
Hydroxytyrosol sulphate (HTS)	0/6 ^a^	5/6 ^b^	0/6 ^a^	3/6 ^b^	1.00	<0.001	0.39
Alcohol homovanillic sulphate (AHVS)	0/6 ^a^	4/6 ^b^	0/6 ^a^	4/6 ^b^	1.00	<0.001	1.00
Both AHVS and HTS	0/6 ^a^	4/6 ^b^	0/6 ^a^	2/6 ^b^	1.00	<0.001	0.35

^1^ Within each row, a different letter (a, b) denotes statistical differences across treatments (*p* < 0.05). ^2^ ADG during the prepartum period was calculated with the BW of the gestating cows, including gravid uterus and conceptus. The BW of the cows after calving did not consider the conceptus or placenta weight. ^3^ HT metabolites in serum were not detected in any cow at week −12 before calving.

## Data Availability

All of the data is contained within the article and the Supplementary Materials.

## Supplementary Materials

The following supporting information can be downloaded at https://www.mdpi.com/article/10.3390/antiox14091097/s1. Table S1: Ingredient composition of the Total Mixed Ration (TMR) (g/kg of diet, as-fed basis); Table S2: Chemical composition and nutritive value of the Total Mixed Ration (TMR) (g/kg DM, unless otherwise stated).
