# Supplementary material for: Maternal Hydroxytyrosol Supplementation Enhances Antioxidant Capacity and Immunometabolic Adaptations in Nutrient-Restricted Beef Cows and Their Offspring
Source: Antioxidants (Basel). 2025 Sep 8;14(9):1097. doi: 10.3390/antiox14091097 (PMC12466888; doi:10.3390/antiox14091097)
Supplement: Supplementary file 1 [file antioxidants-14-01097-s001.zip › antioxidants-3805456-supplementary.pdf]

Supplementary material

Table S1. Ingredient composition of the Total Mixed Ration (TMR) (g/kg of diet, as-fed basis).

Ingredient	Proportion (%)
Barley straw	49.6
Barley grain	24.8
Alfalfa pellets	8.4
Rapeseed meal	6.9
Sugar beet pulp	4.5
Soybean meal	2.5
Calcium carbonate	0.80
Dicalcium phosphate	0.25
Sodium chloride	0.25
Vitamin–micromineral premix	0.20
Water or HT solution[1]	1.8 (18 L/t)

[1]HT solution prepared at 10 g HT/L and incorporated as mash (18 L/t of diet). The control diet received the same amount of water.

Table S2. Chemical composition and nutritive value of the Total Mixed Ration (TMR) (g/kg DM, unless otherwise stated).

Item	Value
Dry matter (g/kg feed)	883
Crude protein (CP)	114
Starch	203
Neutral detergent fiber (NDF)	500
Acid detergent fiber (ADF)	301
Acid detergent lignin (ADL)	48
Ether extract	21
Net energy for lactation (NEL)	1,240 kcal/kg DM
